# A Nature-Based Vocational Training Programme for Migrants and Swedes: Impacts on the Five Ways to Wellbeing

**DOI:** 10.3390/ijerph22081252

**Published:** 2025-08-10

**Authors:** Liz O’Brien, Ann Dolling, Marcus Hedblom, Anna María Pálsdóttir

**Affiliations:** 1 Society and Environment Research Group, Forest Research, Farnham GU10 4LH, UK; 2 Department of Forest Ecology and Management, Swedish University of Agricultural Sciences (SLU), 901 83 Umea, Sweden; ann.dolling@slu.se; 3 Department of Urban and Rural Development, Swedish University of Agricultural Sciences (SLU), 750 07 Uppsala, Sweden; marcus.hedblom@slu.se; 4 Department of People and Society, Swedish University of Agricultural Sciences (SLU), 234 22 Alnarp, Sweden; anna.maria.palsdottir@slu.se

**Keywords:** health, wellbeing, forests, migrants, immigrants, nature-based integration, nature-based intervention

## Abstract

Nature-based interventions are increasingly used to support human wellbeing, and more recently to integrate migrants into host countries. In this research, we focused on a nature-based vocational training programme led by a partnership of three Swedish public organisations. In the programme, long-term unemployed Swedes worked with migrants in various nature conservation and nature management tasks as part of an employment programme. We undertook interviews with nineteen participants and carried out observations ‘in situ’ to explore the impact of the programme on participants’ wellbeing. Using the ‘five ways to wellbeing’ as a conceptual framework, we found that the programme connected participants to nature, helped them take notice of the forests and nature they worked in, and connected participants across cultures. The participants learned new conservation skills and migrants had a chance to improve their Swedish language skills. The activities within the programme were physical and the majority found this was important for their overall wellbeing. Migrants were younger and keener to learn new employable skills than Swedes who were closer to pension age. The groups were more similar when it came to identifying the impact of the programme on their wellbeing. Nature-based vocational training programmes not only allow participants to gain skills for employment but can have a significant impact on wellbeing.

## 1. Introduction

Nature-based solutions and nature-based interventions are being increasingly used for a variety of purposes to support different sections of populations’ health and wellbeing in a range of countries [1]. These targeted populations may be faced with mental or physical ill health, social isolation or exclusion for a variety of reasons [2]. Nature-based integration programmes have also been created to focus on how migrants can be helped to assimilate into host countries [3]. Migrants can face many mental health issues based on their experiences in their home countries, their journeys to seek refuge and a new life elsewhere, and the integration policies in their new host countries, which may offer different levels of support and opportunities for work [4]. However, Alegria et al. [5] suggest that while migrants face several mental health risks, they often have better mental health than expected. They argue that the development of social networks and relationships can support migrants as they settle into their host country. A Swedish report showed that migrants’ self-assessed wellbeing was high in Sweden compared to other countries in Europe [6]. Public attitudes towards migration, were also found to be more positive in Sweden than in other European countries. The report shows a positive correlation between migrants’ wellbeing and public attitudes towards migration, with Sweden and the other Nordic countries among those with both high levels of wellbeing for migrants and high levels of acceptance of migrants [6]. Bartram [7] found migrants to be mostly happier than those who stayed in their home country, while Hendriks and Bartram [8] suggest the social climate of the host country is important for the wellbeing of migrants.

Sweden is one country that took in large numbers of migrants in 2015 during a crisis that was characterised by many migrants, from often non-European countries, travelling to Europe [9]. It is suggested that migration is fundamental to globalisation processes. In Sweden, migrants and refugees came from a range of countries including Syria, Afghanistan, and Iraq. The asylum laws were tightened in Sweden in 2021, and numbers since then have reduced. The increase in migrants into Sweden impacted Swedish society with a Eurobarometer survey showing that just over 50% of the population thought immigration was the most important political issue in 2014 [10]. Public debates about integration were frequently a topic of conversation in the media and linked to concerns about lack of housing, lack of regular jobs for migrants, shortages of teachers, and access to Swedish language courses [11]. According to the World Health Organisation the experience of migrants is a key determinant of their health, and they often face discrimination, poor housing and jobs, and physical and mental health issues [12]. However, a longitudinal cohort study in Sweden found that there was a healthy migrant effect for some particularly Western migrants [13]. Historically since the 1970s Swedish governments have developed integration strategies to foster the inclusion of migrants. A new law in 2016 brought about major change to the Swedish asylum policy. Those gaining international protection would no longer obtain permanent residence but a shorter permit. In 2021, the ‘permanent amendments to the Alien Act’ came into force with all new residence permits being temporary, usually two years. To remain in Sweden, adults need to show how they will support themselves [11,14]. The Swedish Employment Service (Arbetsformedlingen) runs the two-year integration scheme for migrants/refugees that have received asylum in Sweden. The Establishment Programme (Etableringsprogrammet) includes courses on learning the Swedish language, coaching on how to gain employment, and civic orientation to learn about Swedish society [11].

### 1.1. Nature-Based Interventions Aimed at Migrants

A review of nature-based integration of migrants found that aspects that support integration include social interaction and a sense of belonging, and that activities in nature can have positive impacts for migrants [15]. Natural environments and nature-based integration activities can support the learning of different cultures and impact the health and wellbeing of migrants [3]. Charles-Rodriguez et al. [16] looked at migrants’ wellbeing, physical activity and integration and found connecting with pre-migration experiences and social interaction could promote their wellbeing. A small number of Turkish women migrants in Denmark were engaged in a nature-based participatory action research project. They undertook a range of activities in two forests including yoga, meditation and picking berries. All of the participants faced mental and physical health challenges [17]. Social connectedness, feeling calmer and having sore muscles were benefits identified by the women of engaging in the project. The focus on berry picking linked them to their home country and experiences of growing up there. A study of migrant women in Norway asking about their relation to nature and nature experiences had similar outcomes, as the one in Denmark. Women in Norway felt calm in nature and gained mental health improvements and improved social interactions, and this promoted immediate wellbeing and provided future social support [18]. In Canada, migrant families accessing nature gained benefits in their health and wellbeing trajectory, personal receptivity, and local mentoring [19]. While Ramburn et al. [20] reviewed community garden interventions for refugees and migrants and found consistent themes highlighting that these interventions can improve wellbeing and self-worth and can provide a sense of meaning and social connectedness. They argue that community gardens can be important psychosocial interventions and can be one of the services that support migrants. However, experiencing urban green areas in big cities (Berlin and London) without guidance on where to go or how to behave migrants felt was unsettling and potentially risky for them [20]. Ekstam et al. [21] explored migrants’ experiences of a nature-based vocational rehabilitation programme in Sweden. Participants carried out activities in a garden environment, which became seen as a place of refuge and safety where they could let go of everyday worries, even if only for a little while.

### 1.2. Nature-Based Vocational Training Programme and Agency Partnership in Sweden

An innovative partnership was developed in southern Sweden between the Swedish Forestry Agency (SFA—Skogsstyrelsen), the Swedish Public Employment Service (SPES—Arbetsformedlingen) and the County Administrative Board in Skåne (CAB Länssyrelsen). All three organisations have worked together for many years on vocational training programmes for unemployed people [22]. In 2015, they decided to work in partnership to develop a new vocational programme that included migrants within the Establishment programme run by SPES, and long-term Swedish unemployed people. The goal of SPES is to introduce its clients to work and vocational training programmes. SPES offer a range of different options for work and vocational training. The nature-based vocational training programme is one of many options that clients can choose from. All participants can choose based on their own interests, and if they are not satisfied, they can leave the programme at any time. The Swedes have to be long-term unemployed before joining the programme—in Sweden this is classed as those over 25 years of age who have continuously been registered as unemployed for 12 months or 6 months if under 25. Through collaboration with SFA, there is a long-standing offer for anyone listed at SPES to enrol in nature-based vocational training. Swedish long-term unemployed, can enlist for two years of vocational training, and since 2015, migrants can join for 12 months as part of their 24-month integration scheme, this is provided to refugees granted asylum in Sweden. When migrants are granted asylum in Sweden, they are included in a two-year integration programme that provides various activities to support their integration into society and the labour market. Among others, they are offered vocational training in multiple occupations or trades, based on their interests and previous occupation, and Swedish language lessons [23].

**Figure 1:**
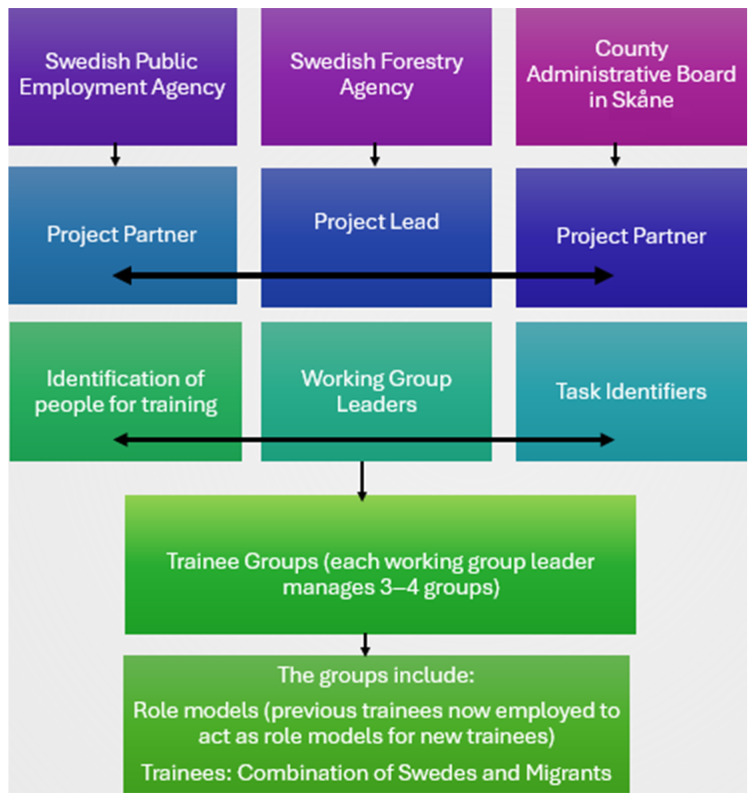
The organisational partnership delivering the nature-based vocational training programme (Slightly revised from [22] with permission).

The programme involved migrants and Swedish unemployed working together in small teams across Southern Skåne to undertake conservation tasks identified by the CAB. The Forestry Agency SFA managed the teams on the ground and SPES identified participants who were interested in getting involved and could benefit from the programme, while CAB helped to identify the sites for the participants to work on (Figure 1). Participants were split into groups of approximately 8–10 people, with each group having a group leader (employed by SFA) overseeing 3–4 groups (a total of 19 groups in the programme). Each group has a local working station as its base camp, where tools and equipment are stored, as well as a place for rest and lunch breaks. The groups undertook a variety of management and conservation tasks, gained practical conservation work experience, and were trained to work towards gaining certificates in chainsaw use and brush cutting. Migrants worked for 3 days a week, with the other 2 days set aside for Swedish language classes, and they spent 1 year on the programme. The long-term unemployed Swedes worked five days a week and spent 2 years on the programme. All participants are paid a wage to participate.

### 1.3. Theoretical Framing: Five Ways to Wellbeing

In this paper we use the five ways to wellbeing as a conceptual framework to explore our data. This framework was developed in the United Kingdom (UK) by the New Economics Foundation. A major Government Office for Science [24] report on mental capital and wellbeing explored how society in the UK could prosper and flourish in terms of its mental wellbeing. The report (called a Foresight report) drew on the evidence and advice from over 400 experts. Within the project the New Economics Foundation (NEF) was asked to review and produce a wellbeing equivalent to the health advice of ‘five a day’. In the UK five pieces of fruit and vegetables a day are recommended by the Government based on advice from the World Health Organisation [25]. Based on its review NEF developed the five ways to mental wellbeing [26] which include: 1) Connect, 2) Be active, 3) Give, 4) Keep learning, and 5) Take notice (see Appendix A). ‘Connect’ is about relationships between people, whether friends, family, colleagues or neighbours and is focused on building positive relationships with others. ‘Be active’ is centred on movement and physical activity, while ‘Keep learning’ is about trying something new, taking up an interest, or learning something new. ‘Give’ is about doing things for others, volunteering, sharing expertise, and finally ‘Take notice’ is linked to concepts of mindfulness and the importance of taking notice of and appreciating the world around you in the current moment. It is suggested that these ‘five ways’ can improve personal mental wellbeing. The framework is used in health and social care [27] by mental health charities [28] and in the National Health Service [29] in the UK. The five ways to wellbeing has been used as an analytical tool and framework in several academic studies, for example, by Stadler et al. [30] to explore wellbeing in the events industry, by Rose and Riley [31] to identify how zoos and aquariums can better connect visitors to promote positive nature experiences, and by Coren et al. [32] to assess volunteers and contributors’ wellbeing connected to a folk week festival. The various studies found the five ways framework to be helpful in accessing their data and framing their results. Therefore, we use the framework in this paper and explore the following research questions:

1. Did the nature-based vocational training programme have an impact on participants perceived wellbeing?
2. Were wellbeing benefits experienced differently by Swedes and Migrants?

## 2. Materials and Methods

The study was conducted as a retrospective semi-structured interview study with trainees in the nature-based vocational training programme. It also included field visits, participant observation and informal discussions with trainees in the field. Semi-structured interviews were undertaken with trainees at the end of their participation in the programme. They were from three different cohorts, i.e., 2016, 2018, and 2019. All trainees received written and oral information about the study presented by one of the researchers visiting them onsite (field visits). Those interested could notify their group leader to make an appointment with the researcher. Those who agreed to participate provided their informed consent. Most of the interviews were conducted at the trainees’ local workstation (i.e., the place they met each day to be given tasks to undertake which often included travel to a specific site) around Skåne County, enabling those who wanted to participate in the study to do so.

We aimed to interview a mix of Swedes and Migrants as well as those in different age ranges. In total, 19 interviews were undertaken, 4 from Swedes and 15 from migrants. Of those, 16 were men and 3 were women, aged between 22 and 65 years. The majority of migrants were younger than the Swedes who tended to be older, i.e., in their fifties and sixties [33]. Migrants included those from Syria, Somalia, Eritrea, Iran and Afghanistan, and most had been granted asylum within the last two years of enrolment. Only information on gender and country of origin was collected. Data on specific age, marital status, duration of unemployment prior to enrolment in the programme, education, and residence was not collected as this was beyond the scope of the study. The interviews lasted from 30 min to an hour. Interpreters were used in interviews with migrants covering six languages: Arabic, Tigrinya, Farsi, Pashto, Somali and Swedish. All the materials about the research and consent were translated into the appropriate language of the interviewee. Eighteen interviews were recorded with the consent of the interviewees; however, one person did not consent to be recorded. Thirteen of the recorded interviews were transcribed, but five proved too difficult to transcribe, and, therefore, the recordings were listened to, and notes were taken. Interview questions focused on how the participants in the programme were experiencing it, what type of activities they were undertaking, what they were learning, and any skills development, as well as any specific training they were receiving that could lead to a certificate. Perceptions about nature and connections to nature as well as perspectives on working in a team and across different cultures were also included.

Pálsdóttir undertook the interviews and the fieldwork travelling to different parts of the county to visit all the groups on two occasions. This provided an opportunity to observe what the groups were performing, the activities they were undertaking and how they worked together. It also provided opportunities for short discussions with the group supervisors and with the workers in each group. Observing the practical everyday practices of the groups and how they worked together provided a rich further insight into the programme and its participants and how they worked as teams undertaking a variety of tasks.

### Data Analysis

The majority of interviews were transcribed verbatim. Notes were taken after each field visit from the observations made. The coding included deductive and inductive approaches (Table 1). The five ways to wellbeing were used as a broad deductive framework for the analysis. All interviews were treated as a single set of data, and no comparisons were made between genders or countries of origin; due to the size of the dataset this was not feasible. Each transcript was read and re-read to familiarise ourselves with the data and the interviews not transcribed were listened to again. We then allocated sentences to one of the five ways to wellbeing or allocated then to an ‘other’ code that was then read and coded. After the deductive coding we developed inductive codes that we identified as themes in the data. The authors reflected and discussed each theme and its content. Each broad wellbeing category could be further coded, for example, connect was split into the concept of connecting to nature and connecting to people. “The importance of work” to the participants was identified as a new higher-level theme in the data and had connections to the ‘five ways to wellbeing’ theme of ‘learning’ but was different enough to stand alone as a theme. We followed the broad thematic analytical approach of Braun and Clarke [34], which focuses on and interprets the experiential realities of participants.

## 3. Results

We present the findings using the five ways to wellbeing as a deductive framework and also include other themes that have arisen inductively from the data.

### 3.1. Connect

#### 3.1.1. Connect to People

There were a range of ways in which participants in the programme could connect with each other. Carrying out forestry work in a team was generally viewed as a positive experience. One migrant said his teammates were very kind and had become good friends, he suggested:

*‘It feels great with the comrades and the surroundings’ (Male, Syria, Interview 1)*

Another talked about the importance of socialising within the team and getting to know others both on a personal and on a cultural level. The teamwork enabled them to meet and bond with each other in a way that they might not normally do in everyday life. Friendships were made across the groups.

*It’s great to get to know other ethnic groups, also being mixed a bit *[Swedes and migrants]* and finding mixed friends’ (Male, Afghanistan Interview 6)*

However, there could be problems with one person outlining that some in the team did not always do the work they were supposed to do properly. This seemed to be an issue of differences in language, with some migrants mainly speaking in their native language rather than trying to speak Swedish. This was particularly the case in teams that had only one native Swede as part of the team. However, one Syrian was very keen to work with Swedes to improve his language skills. While another migrant wrote a diary of his activities specifically to improve his Swedish.

A female migrant wanted to continue to work with the forestry programme because she has made new friends. She also talked about feeling safer than in her home country and more secure in Sweden. A younger migrant said the best part of the programme was meeting people and the camaraderie. He has also introduced others to nature, pointing out birdsong to his companions. He also brought friends to the nature reserves where he worked to show them the attractive places they could visit:

*“I showed them a little, we bring coffee and (…) then we talked a little, then back to Malmö.” (Male, Somalia, Interview 12)*

An older man from Syria said that he often talked to elderly people who were walking their dogs when he was going about his work, and this connected him to local people who would sometimes ask questions about what the teams were trying to do.

*“Sometimes, some people stop and talk to us. A little Swedish. I explain that I know a little Swedish and they try to speak slowly.” (Male, Syria, Interview 13)*

A Swedish woman who has been part of the project off and on for about 10 years, has worked with some participants for many years. She outlined that the project involved Swedes only in the beginning, but more migrants have joined in recent years. She explained that the migrants she worked with have been hardworking and eager to learn Swedish. She believed the project was an excellent way for migrants to improve their language skills. She described how they look after each other, they have coffee breaks and lunch together, and if someone withdraws, the group notices and tries to bring them back into the group chat:

*…”then we try to pull them in, we have done that anyway.” “And if there is someone who might rather be outside than sitting in the booth, then maybe you take your coffee cup when you have finished eating and go out and talk.” (Female Swedish, Interview 10)*

#### 3.1.2. Connect to Places/Nature

The majority of interviewees valued working in the forest environment. One migrant from Syria outlined that it was his first time being out in a forest when he started on the programme and he really enjoyed the impact on his wellbeing and highlighted its importance by suggesting:

*‘In the forest you feel very easy and it’s rehabilitation’ (Male, Syria, Interview 1)*

Another man talked about his connection to nature from growing up in Sweden. He had managed some cows and had enjoyed eating from nature picking fruits such as apples and plums. He also enjoys the winter in the forest in Sweden and forages for nettles, which he outlined were good for people. He stated the following:

*‘I thank the forest it has given me a life, a very good life’ (Male, Sweden, Interview 4)*

Interviewees talked about enjoying working outdoors, sometimes they talked about different types of nature and particularly how one type of nature can help them feel more connected. For example, one man talked about liking forests more than meadows because of how they make him feel which includes coping with his current situation, as his interpreter outlined:

*‘He finds the trees are calming, he feels good and it makes him confident so that he can handle life (Male, Syria Interview 2)*

The migrants talked about liking to work outdoors. One of the women felt satisfied and healthy due to being outside and experiencing nature, which also gave her a sense of freedom, which can be quite common for those working in nature:

*“Yes I like to work… you are free.” (Female, Syria, Interview 9)*

*Great freedom *[from being outdoors],* nature is like a vitamin for me (Male, Syria, Interview 8)*

One of the migrants talked about putting down roots in Sweden and feeling comfortable in the country, while another talked about feeling at home and that Sweden was now his home country and part of his identity:

*‘I feel I am Swedish and my roots should be stuck here. I don’t want to move back there *[Syria]* (Male, Syria, Interview 2)*

Several participants described how they enjoyed the forest and wider nature beyond the forest and the importance of nature to them, and for overall human survival.

*“A lot, so nature is a force that we never, so if we hadn’t had nature we would never have, we would have found it difficult to exist at all if nature hadn’t existed, but our trees and such, yes they clean the air, the oceans, yes they help us make sure on that point so that if we destroy nature, it’s over.” (Women, Swedish Interview 10)*

Participants also talked about connecting to nature and forests by observing wildlife or just sitting to take in what was around you, and they could find peace in nature as the following quotes suggest:

*“Take a cup of coffee with you and go out and sit somewhere on a stump, there’s nothing better.” (Female, Sweden, Interview 10)*

*“The calm actually. It’s quite nice to see and sit and just like yes, birds and everything.” (Female, Sweden, interview 10)*

A male migrant had not initially considered working in the forest, but after participating in a project at the rehabilitation garden in Alnarp, near Malmo, he became more interested in working in nature. He had experience of spending time in the forest and cultivating land privately in and around his home in his native country. But at first, he was not interested in turning it into a job. The man has since continued working in the forest and says that being in the forest makes him feel content:

*“A few days ago I went to sign an employment contract. And when the employer shows me the nature or the forest where I will be working, I can tell you that I felt very good mentally and I have become completely calm when I saw nature as well.” (Male, Syria, Interview 11)*

He also described a special connection between himself and the trees:

*“It’s something difficult and big and tall trees and something like… It’s like something challenging between me and the trees.” (Male, Syria, Interview 11)*

### 3.2. Learning

The majority of participants were keen to learn, and this was primarily associated with obtaining a job and gaining employment. The migrants felt a strong sense of pride in having a place on the programme and being able to work.

*I thought it was fun to be in nature and learn a lot about trees and nature… it was special (Male, Afghanistan, Interview 6)*

A migrant spoke about learning a lot about how to behave as an employee and supervisor as he has previously been self-employed. One Swedish man wanted to apply for the programme again but recognised that the rules meant he could not do this for 2 years after he had completed his current placement. He was a group leader and outlined how it was a responsible job for him. One of the things he learnt about different cultures was that during Ramadan the migrants found it more difficult to undertake some of the more physical work as they were not eating between sunrise and sunset. One of the migrants was curious about Swedish mid-summer celebrations and was keen to know and learn more. Another Swedish man talked about learning on the job and about passing his practical chainsaw course but not the theory element of the course, which he said was very difficult.

One of the Syrian men was keen to further develop himself and wanted to gain the practical experience that the programme offered. This was similar to another Syrian man who wanted to learn how to use a chainsaw and watched videos about this in his spare time to accelerate his learning. A Swedish group leader talked about the fun of learning on the job and how this could enable participants to become more confident and comfortable in what they were doing. The Syrian man whose quote is outlined below commutes to the job for about 4 h per day and is keen to work most days. He also outlined that he works to know and learn more about himself and what he is capable of, not only about the particular job he is undertaking. This learning helps him to deal with what life throws at him.

*‘I feel confident more, I feel that I am, I can handle life. It’s a shame that they didn’t work on Saturday and Sunday as well’ (Male, Syria, Interview 2)*

Through the work the participants, both migrants and Swedes talked about practical learning how to put up fences, repair bridges, fix ladders, cut grass, fell trees, use chainsaws and brush cutters, and learn to look after their tools. There were also opportunities for language development and one man stated that he liked to work with his Swedish colleagues so that he could practice speaking in Swedish. Many of the participants obtained brush cutter and chainsaw certifications. They highlighted that these certifications were some of the most important skills they have learned while participating in the programme. One migrant was motivated to study independently to obtain the proper qualifications to operate a chainsaw:

*“And then at school they changed it that you have to have a C to work with a chainsaw. And when I discovered that I don’t have the right according to the school to take this chainsaw license so I took the book with me and started (…) chainsaw and then I started studying at home and so on my own, I did.” “I can also tell you that the first time I failed the theory but I decided not to go home and studied all night until the next day. The next day I passed, 20 out of 20.” (Male, Syria, Interview 11)*

One of the Swedish women believed that the migrants learnt both the Swedish language and how Swedish society functions while on the programme. A young migrant who has been in Sweden for four and a half years has learned Swedish so well that he now acts as a language support for others. One of the Swedes talked about learning a few words of another language to connect with others in the group he worked in, and he also talked about helping other to learn Swedish words.

### 3.3. Take Notice

Participants took notice of the surroundings in which they were working and, therefore, this theme is closely linked to the theme of connecting to place/nature. One migrant talked about liking fresh nature and enjoyed the smells of the outdoors; he said he was happy in the forest and suggested the following

*‘Sweden’s nature is beautiful as a god’ (Male, Syria, Interview 1)*

Another talked about liking to be out in the forest and to learn about new tree species. Migrants talked about the differences between the nature they were used to at home and the landscape of Sweden. They sometimes talked about the harsher weather in Sweden compared to their home countries. One man in the beginning had difficulties with the cold, rain and wind when working outdoors. However, once he obtained protective clothing, he felt much better able to cope with the different types of weather. A Swedish woman described the job as varied and said it allowed her to move through different types of nature that she enjoys:

*“The forest is actually nice. After all, meadows are also… beautiful so that there are many different types of nature.” (Female, Sweden, Interview 10)*

A young migrant described how he wanted to work outdoors because of the fresh air and greenery:

*“Every day you see everything green, when it’s not winter, but when it’s winter all the leaves come down… It’s good when it’s autumn too.”(Male, Somalia, Interview 12)*

Another talked about noticing a lot about how the seasons differed between Sweden and Syria, with more defined differences between the seasons in Syria than in Sweden, where there can be four seasons in one day. He also highlighted it was very much cooler or there was cold weather more of the time in Sweden. A man from Afghanistan mentioned that the air seemed cleaner in Sweden and there were more trees and fewer mountains than in his home country. While one of the Swedes talked about noticing and recognising how difficult it can be for migrants to get used to a new country, language and ways of working.

### 3.4. Be Active

The whole programme is focused on practical conservation activities and, therefore, the majority of what the participants were engaged in was physical work. Many highlighted that there were benefits of being physically active and it helped them to feel alert and healthy. It could also stave off illness as one person said they had not had a day off sick since starting work. One of the migrants talked about becoming hot and sweaty from the physical work they undertook, and this was part of his identity, he stated the following:

*‘You sweat you are somebody’ (Male, Syria, Interview 1)*

A woman from Syria stated that she drives a chain saw, a clearing saw, cuts trees, removes birch, collects brash, makes fires and also makes fences: all of these are physical activities. She says that it becomes a habit to engage with physically heavy work and has no problem with this. While a man from Syria talked about throwing himself into his work and how in his home country, he worked just to survive but in Sweden he is doing more than just surviving.

A Swedish older woman who has been involved in many activities highlighted the physicality of the job and how it is linked to health and weight loss for her, she stated that:

*“Overall, you move a lot. It is physical, very physical. You don’t need to train anything else.” “Yes, there is a lot, a lot of health. When I started this job in 2004, I was overweight. I lost 18 kg, 18 kg I lost in a one-year period”. (Female, Sweden, Interview 10)*

She suffered from severe migraines when she worked in an office, but after starting physical work in the programme, her migraines are now completely gone:

*“I think it’s because you’re out and physically moving.” “It’s a different focus, you focus with your whole body instead of just your eyes.” (Female, Sweden, Interview 10)*

A young man also described the work as physically demanding, comparing it to being at the gym all day:

*“For me it’s physical work, you can say it’s a gym so you take that machine, you work on it, you take a break and (…) you get to work on a second machine, we help each other.” (Male, Somalia, Interview 12)*

One migrant also outlined that his Swedish language skills were not too good, and he found this stressful. His children were becoming good at speaking Swedish, but he was struggling, however he could forget about this when out working in the forest and undertaking hard physical activity.

### 3.5. Give

One migrant had shown the forests and nature reserves where she works to her friends, and they had been there for a barbecue and to look at what she described as ‘beautiful nature’. Another migrant talked about working in a park, which attracts visitors from all over the world; it highlighted to him the importance of the work they do, which provides benefits and enjoyment to visitors. A Swedish supervisor outlined the need to have patience and calm when explaining the jobs that are needed to be performed by others.

One man shared how schoolchildren visit the nature reserves, and those who are working have to explain what they are doing and why:

*“You kind of have to get to the children’s level, i.e., the children’s thinking. We say the tree is sick, we have to burn it and why.” (Male Syria, Interview 13)*

Others talked about helping each other out with language skills, with some of the tasks and activities as well as looking out for each other when someone was having a bad day or a difficult time. The camaraderie of the teams was spoken about by participants as important along with the cross-cultural connections.

### 3.6. Importance of Work

A Syrian man talked about being a little scared on joining the programme because he was unsure of exactly what the work would entail but once he got involved and grew to understand what was required, he enjoyed the work and was very sorry when it ended. He outlined that this was the sort of job he would like to have permanently. While another participant said that due to the work, he undertook he became a person who was needed and this gave him a sense of satisfaction and meaning. Most participants were keen to work, and they gained pride in the job they had and the work they were undertaking. One Swede said work makes him feel like a real man, while a migrant outlined how work can give people a sense of self-worth and help you to feel that you are somebody. Another stated:

*‘It is like a miracle *[to be employed in Sweden]* (Male, Syria, Interview 2)*

Another participant stated that he feels like a different man through working on the programme, it is very important for him to be working in Sweden, and he feels a sense of worth and identity from the job he has. A couple of the Swedes were in their early sixties and wanted to retire in their mid-sixties but talked about how regulations concerning retirement were an issue in Sweden. One wanted another two years of work before retiring but wanted to stay and do work in the forest. However, his time on the programme was nearly at an end and he would have to look for something else. Participants who have been on the programme cannot immediately become involved with it again; they have to wait two years before they can apply to join again. This participant was not hopeful of finding something suitable that he could do. Another Swede talked about how having a wage was a big motivation and it made him feel like he had a real job, rather than someone on a time bounded programme, he also felt stronger because he was working and that was not just in a physical sense.

An older man shared that he has diabetes, yet he is never sick or takes time off. When he has an appointment with a doctor or dietitian, he tries to reschedule it for the afternoon so he can go after work has finished. He was focused on his responsibility as someone who drives others to the workplace:

*“If I have to take time off, there are 7 others who have to be off, there is no other driver who can drive them, so no other option.” (Male, Syria, Interview 13)*

A Syrian men talked about how he had worked in his home country but also in Lebanon to be able to earn enough for food, but now he worked for more than that in Sweden. He said he worked to live and to know himself more and this gave him an important sense of satisfaction.

## 4. Discussion

In this study we explored the impact of a long-term partnership between three public Swedish bodies that have collaborated and developed an innovative programme bringing together migrants and long-term unemployed Swedes to carry out nature conservation tasks and how this affected the participants wellbeing.

### 4.1. Difference and Similarities Between Swedes and Migrants

The migrants tended to be younger in age than the Swedes, and they were very keen to develop their skills and gain full-time employment after their time on the programme had ended. Some of the Swedes were older and near retirement age and were looking for another couple of years of work before they could officially retire. All enjoyed the physical aspects of the job with some outlining weight loss, fitness improvements and stress reduction that can come with physical activity. All were pleased to be out in nature, with migrants outlining the differences between the landscapes in their home country and Swedish nature in terms of aesthetics, different types of habitats, vegetation cover, trees, and temperature, as well as differences in seasons and overall weather patterns. These could be appreciated but also could take some time for migrants to get used to. All gained a sense of pride in having a job and doing something meaningful—managing forests and nature for conservation and for public visitors to benefit from visits to these spaces. Soucy et al. [35] talk about how conservation activities can give people a sense of fulfilment as the work is meaningful and differences can be seen via changes to biodiversity or improved pathways for example.

Learning the Swedish language was a challenge for the migrants, and some were having more success than others. However, working in mixed teams and having short chats with members of the public visiting these nature spaces could support practice and learning. The Swedes learnt a bit about other cultures and cultural practices such as Ramadam which could help them to understand their colleagues better. The migrants were learning about Swedish culture, nature and norms. All seemed to feel connected to nature, sometimes this was linked to childhood or previous experiences and sometimes it concerned new nature experiences. Engagement with the programme provided participants with access to forests and nature sites they may never have visited, particularly the migrants. Jay and Schraml [36] in a study of Russian and Turkish migrants in Germany found older Russians nostalgic for the forests of their childhood, but they did not feel the same connection with the German forests and did not visit them. However, the Turkish migrants from an arid landscape in their home country enjoyed and appreciated the forest environment. In this study, the migrants appreciated Swedish nature and some of the migrants took their families and friends to these spaces that they had become more familiar with. This could potentially translate into greater nature connection and/or protective attitudes and behaviours towards nature [37,38,39].

### 4.2. Meaningful Work and Its Benefits

A number of the participants explicitly talked about the benefits of the work and how being out in nature could improve their mental wellbeing, they talked about feeling good, being happy in the forest, feeling rehabilitated and calmer. Migration is a major life event, and it is stressful to leave one’s country and move to a new country with a different culture [40]. Long term unemployment is also stressful, and research has shown that this impacts people’s wellbeing and is not something they get used to. Gedikli et al. [41] in a meta-analysis across different countries found negative impacts on mental health and life satisfaction for those who were unemployed. They also found the effect was larger for men than women and the impact was larger the longer the term of unemployment was. While Martela et al. [42] found that autonomy and beneficence, e.g., working for the betterment of society and having a positive impact on others, made an important contribution to meaningful work for people. Interestingly this highlights that both self-fulfilment and working with others combine for meaningful work. This resonates with nature-based vocational training as most participants found the social connections and interactions, and improving nature spaces that the public can access and enjoy were important. But also, participants wanted to make a personal contribution and outlined that work was crucial as part of their own identity. This was also an important factor of Rai et al.’s [15] review of nature-based integration of migrants. Some studies suggest that engaging with nature can support migrants’ integration [18,43]. In this study participants both migrants and Swedes enjoyed engagement with nature/forest visitors while they were undertaking their work. This allows local people to see migrants and Swedes working productively alongside each other and could potentially support feelings of integration.

### 4.3. Use of the Five Ways to Wellbeing

The five ways to wellbeing framework provided a useful conceptual framework to use to study the participants in the programme both Swedes and migrants and explore their experiences both positive and negative. Participants were overwhelmingly positive about the programme, with migrants primarily wanting to gain skills to find long-term employment and Swedes wanting to work outdoors in the type of work and environment they enjoyed. Negative issues arose around the temperature in Sweden versus the home country of some migrants, people in groups sometimes talking in their native language rather than Swedish and, therefore, not learning the language quickly or well enough to use it in conversation, and the short-term nature of the employment. Our results show that there is an overlap between the five ways. For example, ‘taking notice’ can be linked to ‘connecting to nature’; and ‘connecting to people’ can be linked to the concept of ‘give’. However, we did not find this problematic as it ensured we were better able to explore a multitude of ways in which the programme could impact on the wellbeing of those taking part that might have been missed if we only focused on one type of wellbeing.

### 4.4. Challenges and Limitations of Researching the Programme

Research into the programme raised several challenges and we worked closely with the partner organisations to gain access to participants to explore our research questions. Interpreters were used to cover the six different languages spoken in the programme. However, the meaning of specific words or phrases can be lost in translation. The programme has changed since its inception in 2016 due to changes in the government, the COVID-19 pandemic, and further restrictions on immigration outlined in the introduction. The impacts on participants’ wellbeing were overwhelming positive and there are a number of reasons why this might be the case. Participants were able to make a choice about what programme to become involved in and those that were interested in working outdoors were potentially well disposed to enjoy working in nature on conservation activities. Participants may have felt that they needed to respond positively in case negative comments were fed back to the organisers of the programme and attributed to themselves, even though we outlined that what they said at the interview would be anonymised. We were unable to encourage more Swedes to participate in the interviews. Within the wider vocational programme a number of the Swedes had participated in the programme previously, and at least one Swede in this study had been involved for over ten years (i.e., work for 2 years on the programme and then waiting 2 years before applying again in-line with the regulations for participating in the programme), as they particularly enjoyed this type of work but were unable to obtain a permanent job in this area. They may have felt that their interest in undertaking this type of work was evidence enough that it was important to them. The migrants were proud to have paid work, pay taxes as well as having a paid vacation via the programme. They were eager to stay and build a new life in the country.

We were unable to find out whether participants in the programme went on to gain employment in Sweden. In the current programme there is no link to the labour market and future job opportunities, the participants must look for jobs themselves. Another Swedish project with migrants called ‘Vaxtplats Rosengard’ [44] combined vocational training in construction/building, cooking/catering, and horticulture/urban agriculture, along with training in a workplace. This type of approach can enhance opportunities for participants to gain future employment and could be usefully considered by the current partnership. Visits to some of the teams ‘in situ’ and being an observer provided a very useful way to explore the teams in action and the tasks they undertook, as well as their interactions. The mixed methods approach of interviews and observations can add an extra dimension of insight into participants’ experiences.

## 5. Conclusions

In this study we explored whether participating in a nature-based vocational training programme had an impact on participants’ wellbeing. This innovative programme run in a partnership between three public bodies in Sweden involves bringing together migrants and Swedes to carry out conservation tasks. This study provides evidence that the programme contributed to wellbeing for both groups and that Swedes and migrants were very positive about developing their skills, having a physically active job, being out in nature, learning, and working with others. The five ways to wellbeing was used as a theoretical framework to explore different aspects of wellbeing that are linked to mental health. It provided an opportunity to explore aspects such as connecting with people and nature, learning, taking notice, give, and being active, and an extensive body of evidence has shown that these are all important aspects of wellbeing. The significance of employment for wellbeing has also been outlined in a range of studies providing meaning, self-worth and contributing to identity and wellbeing. Improving wellbeing via meaningful vocational training is an important goal as the long term unemployed and migrants face many challenges but also want to play a meaningful role in society. The participants in the study were very positive about their experiences and we outline why this might be the case such as participants being able to choose what training they became involved in and enjoying being in forests and wider nature. Engagement with nature has been shown in many studies to have a positive impact on people’s wellbeing and particularly so for those facing mental health challenges. Nature-based vocational training such as this provides important opportunities for participants to learn how to carry out a variety of conservation tasks, opportunities for cultural exchange, and understanding that can lead to new cross-cultural friendships. Our study shows that while employment is very important focusing on how it impacts subjective wellbeing provides a more rounded and nuanced view when exploring nature-based interventions. Investing in nature-based vocational training not only supports pathways to employment but also delivers important wellbeing outcomes such as social cohesion and fostering a sense of belonging that is essential for sustainable integration across cultures.

## Figures and Tables

**Table 1:** Deductive and inductive coding of the interview data under key themes.

Higher Level Themes—Five Ways (Deductive) and Importance of Work (Inductive)	Second Level Themes (Inductive)
Connect to people	Making friends Socialising within the team Feeling safe
Give	Connecting family and friends with nature Supporting the team—language and wellbeing
Connect to nature	Getting to know Swedish nature Freedom outdoors
Keep learning	New language and its challenges Other cultures New practical skills
Be active	Physically demanding work Feeling alert and healthy
Take notice	Nature differences between home country and new country Seasonal differences New nature experiences
Importance of work	Satisfaction and meaning Sense of work and identity Working to know oneself

**Figure A1:**
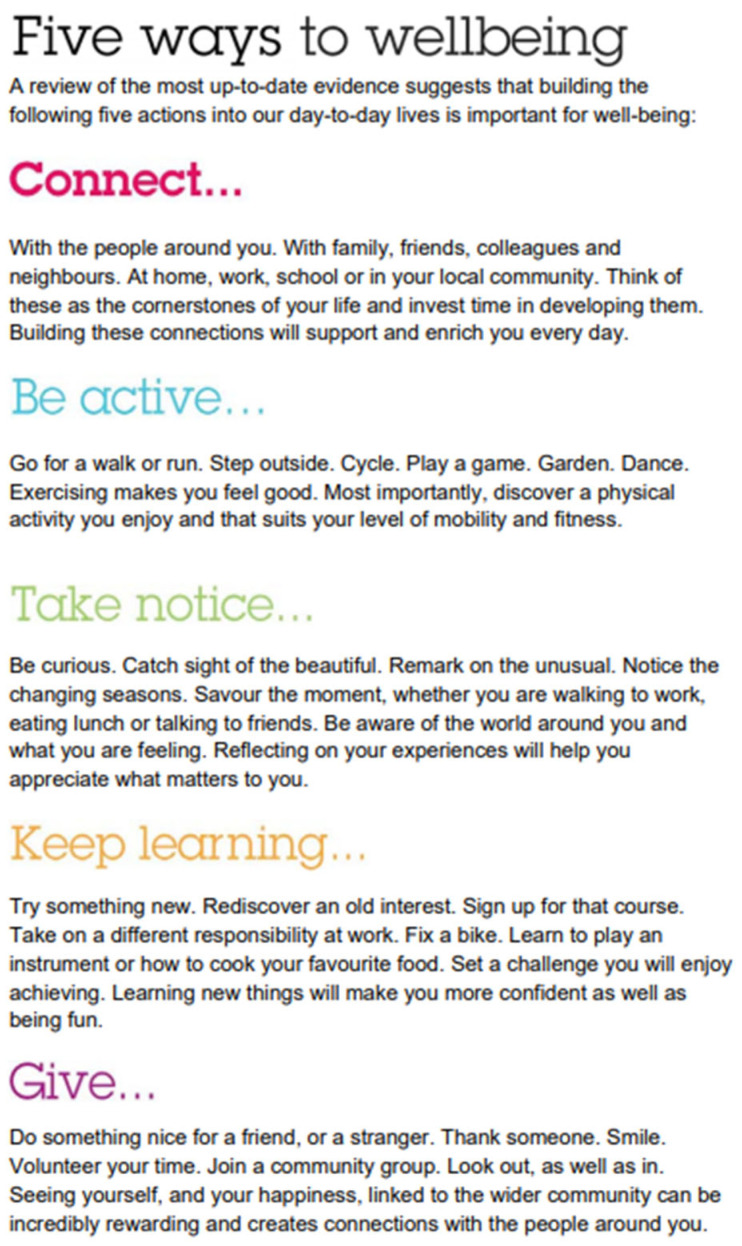
Five ways to wellbeing.

## Data Availability

Due to the sensitive nature of our research the raw data is not available for ethical reasons. The participants did not give clear written consent for the data to be shared publicly.

## Appendix A

Below are the five ways to wellbeing developed by the New Economics Foundation (page 3) https://new-economicsf.files.svdcdn.com/production/files/five-ways-to-wellbeing-1.pdf (accessed on 15th October 2024).
